# Customized Loading of microRNA-126 to Small Extracellular Vesicle-Derived Vehicles Improves Cardiac Function after Myocardial Infarction

**DOI:** 10.1021/acsnano.3c01534

**Published:** 2023-09-16

**Authors:** Sruti Bheri, Milton E. Brown, Hyun-Ji Park, Olga Brazhkina, Felipe Takaesu, Michael E. Davis

**Affiliations:** † Wallace H. Coulter Department of Biomedical Engineering, Georgia Institute of Technology and Emory University, Atlanta, Georgia 30332, United States; ‡ Department of Molecular Science and Technology, Ajou University, Suwon 16499, Korea; § Biochemistry, Cell and Developmental Biology Graduate Training Program, Graduate Division of Biological and Biomedical Sciences, Laney Graduate School, Emory University, Atlanta, Georgia 30332, United States; ∥ Children’s Heart Research and Outcomes (HeRO) Center, Children’s Healthcare of Atlanta and Emory University, Atlanta, Georgia 30322, United States

**Keywords:** Extracellular vesicle, vesicle engineering, nanovesicle, miR-126, myocardial infarction, ischemia-reperfusion injury

## Abstract

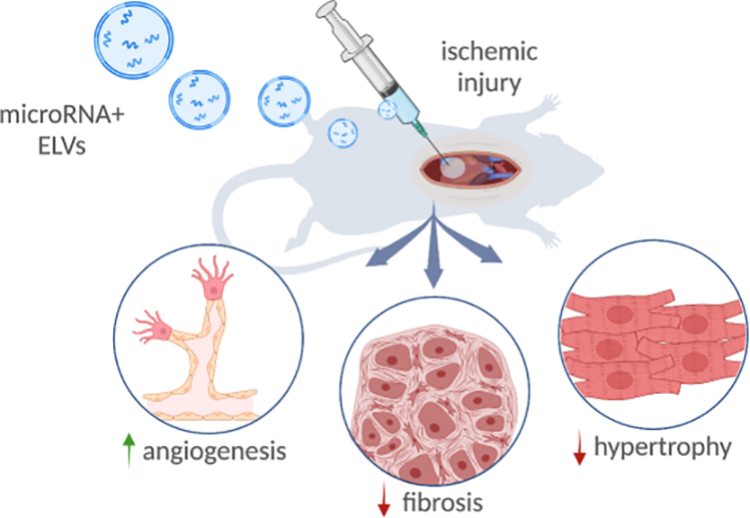

Small extracellular vesicles (sEVs) are promising for cell-based cardiac repair after myocardial infarction. These sEVs encapsulate potent cargo, including microRNAs (miRs), within a bilayer membrane that aids sEV uptake when administered to cells. However, despite their efficacy, sEV therapies are limited by inconsistencies in the sEV release from parent cells and variability in cargo encapsulation. Synthetic sEV mimics with artificial bilayer membranes allow for cargo control but suffer poor stability and rapid clearance when administered *in vivo*. Here, we developed an sEV-like vehicle (ELV) using an electroporation technique, building upon our previously published work, and investigated the potency of delivering electroporated ELVs with pro-angiogenic miR-126 both *in vitro* and *in vivo* to a rat model of ischemia–reperfusion. We show that electroporated miR-126+ ELVs improve tube formation parameters when administered to 2D cultures of cardiac endothelial cells and improve both echocardiographic and histological parameters when delivered to a rat left ventricle after ischemia reperfusion injury. This work emphasizes the value of using electroporated ELVs as vehicles for delivery of select miR cargo for cardiac repair.

## Background

Myocardial infarction (MI) is a major cause of mortality in the United States and commonly results from cardiac tissue ischemia after a coronary artery occlusion.^1^ Clinical trials assessing cardiac cell therapy for recovery after MI show increases in viable heart mass, improved contractility, and reduced scar mass.^2^ Moreover, a trial investigating the combinatorial effect of ckit+ progenitor cells (CPCs) and mesenchymal stromal cells (MSCs) for heart failure found improvements in patient quality of life and major adverse cardiac events.^3,4^ Although these trials involve direct cell therapy administration to patients, small extracellular vesicle (sEV) based signaling likely plays a key role in the observed effects. In fact, studies have found that the improvements observed from stem or progenitor cell therapies might not be directly affected by the cell implantation but rather through the paracrine factors, specifically sEVs, that the cells release.^5,6^

In animal models, sEVs are known to induce cardiac repair when administered after MI.^7,8^ Prior studies in rodent models using MSC- and adipose derived stem cell-sEVs found improvements in cardiac function specifically through improvements in the left ventricular ejection fraction (EF) and fractional shortening (FS).^9,10^ In a porcine model of MI, cardiosphere-derived-sEVs decreased infarct size and promoted neovascularization.^11^ Similarly, in a rat model of MI, CPC-sEVs reduced infarct size and improved EF.^12^ In addition, sEVs have also been studied in human models of Duchenne muscular dystrophy, wherein, priming of induced-cardiomyocytes with cardiosphere-derived sEVs reduced arrhythmogenicity and normalized oxygen consumption rate.^13^ Although sEV therapies in both animal and human models are still in a more nascent stage, particularly those derived from CPCs, these findings show that sEVs can be therapeutic and can have cardiac benefits similar to those from direct delivery of parent stem or progenitor cells.

However, a limitation of these current sEV studies is the extent of the improvements. The *in vivo* therapeutic benefit results in only a roughly 3% increase in echocardiographic parameters.^14^ In addition, the reparative capacity of the sEVs is cell dependent and varies based on CPC conditions such as microenvironmental oxygen levels and parent cell age.^8,15^ Specifically, sEVs derived from younger CPCs and under hypoxic conditions have been more reparative. Further, it is shown that the microRNA (miR) cargo profile of sEV also differs with changes in parent cell conditions. All this suggests that, despite the reparative capacity of CPC sEVs, there is high variability in outcomes. This is reflected across the animal studies, wherein the therapeutic benefits and improvements are inconsistent across studies.

One avenue for this variability is the cargo present within the sEVs. Synthetic mimics on the sEV scale minimize the cargo variability but are rapidly flushed-out when administered *in vivo*.^16,17^ To address the limitations with synthetic mimics but allow for the function of sEVs, we engineered our own sEV-like vehicles (ELVs) from CPC-derived sEVs. Unlike completely synthetic mimics, ELVs likely maintain a similar membrane to that of the CPC sEVs and this could aid in their uptake when delivered *in vivo*. Further, they allow for cargo customizability, especially for large scale cardiac therapies, and thereby could bolster the reparative effects observed from sEVs *in vivo* and minimize the batch-to-batch variations.

In this study, we investigated the therapeutic benefit of engineered miR-126+ ELVs in a rat model of ischemia reperfusion. We show that the ELVs are successfully and controllably loaded with endothelial specific marker miR-126 using electroporation and validate the global and tissue level response of ELV administration after ischemia reperfusion. We observed that miR-126+ ELVs reduce infarct size, fibrosis, and hypertrophy. Further, ELV treatment significantly improved vessel-specific parameters around the infarcted area, which are crucial for recovery after the onset of ischemia. This study underscores the value of maintaining an sEV-like membrane while allowing customizable cargo loading and confirms that the benefits seen with miR-126+ELVs *in vitro* can translate *in vivo* as well.

## Results

### CPC-Derived ELVs Synthesized with Electroporation

**Figure 1:**
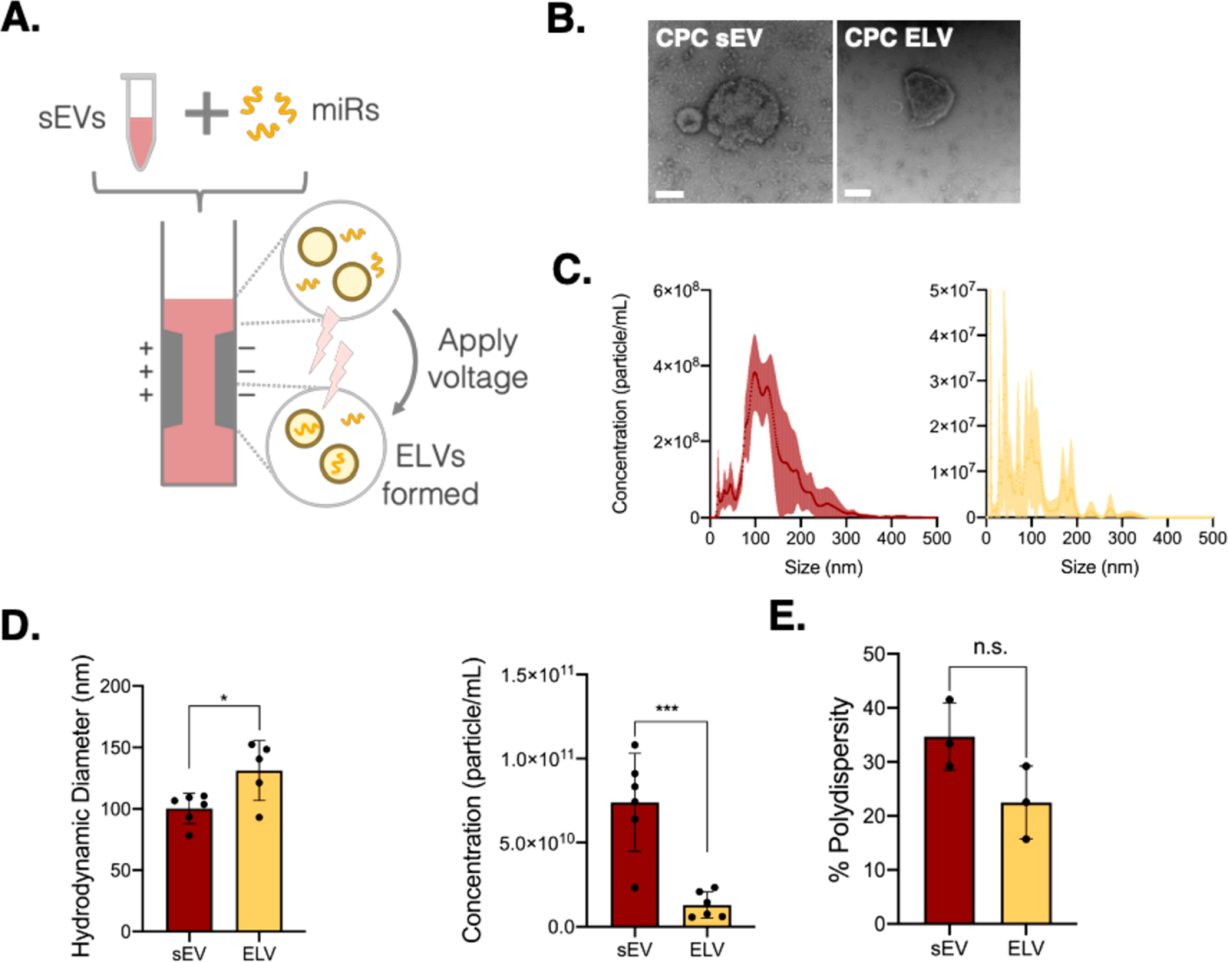
Synthesis and characterization of ELVs using an electroporation method. (A) Workflow of ELV synthesis from CPC-sEVs by sonication-based cargo removal and electroporation-based miR-126 loading. (B) Transmission electron microscopy images of CPC sEV and electroporation-based CPC ELV. Scale bar = 100 nm. (C) Concentration-size profiles of CPC-sEVs and electroporated ELVs measured with NTA. (D) Comparison of ELV and sEV size and concentration. (E) Comparison of percentage polydispersity index of ELVs and sEVs. Mean ± SEM. Significance was tested with two-way Student’s paired *t* test. n.s. = not significant. *P < 0.05, ***P < 0.001.

We explored electroporation as a method for CPC-derived ELV synthesis to build upon our previous work using thin-film hydration.^18^ Electroporation uses small voltage pulses to create temporary openings in the vesicle membrane and allows the miR cargo of choice to be encapsulated into the vesicle through a diffusion gradient (Figure 1A). We confirmed that the shape of the synthesized ELVs was similar to that of CPC sEVs by using transmission electron microscopy (Figure 1B). Comparing the concentration profiles assessed through nanoparticle tracking analysis (NTA), we found that both sEVs and ELVs had similar profiles (Figure 1C). The electroporated ELV sizes were slightly higher than those of sEVs but still within the EV range of 30–150 nm (Figure 1D). Further, the ELV concentration was significantly less than that of sEVs, which is likely attributed to ELV sample dilution during postprocessing, particularly during ELV purification after electroporation, wherein the ELVs undergo a series of ultracentrifugation steps. Despite this, batch-to-batch variation was dramatically reduced with a much smaller deviation (1.30 × 10^10^ ± 7.73 × 10^9^) between batches. These vesicles were also still on the 10^10^ scale, which provided sufficient particles for downstream assessment. Finally, we determined the percentage polydispersity of these ELVs, which was similar to that of sEVs, suggesting an analogous modality in the samples, as well (Figure 1E).

### Electroporation of ELVs Allows for Cargo Tunability

**Figure 2:**
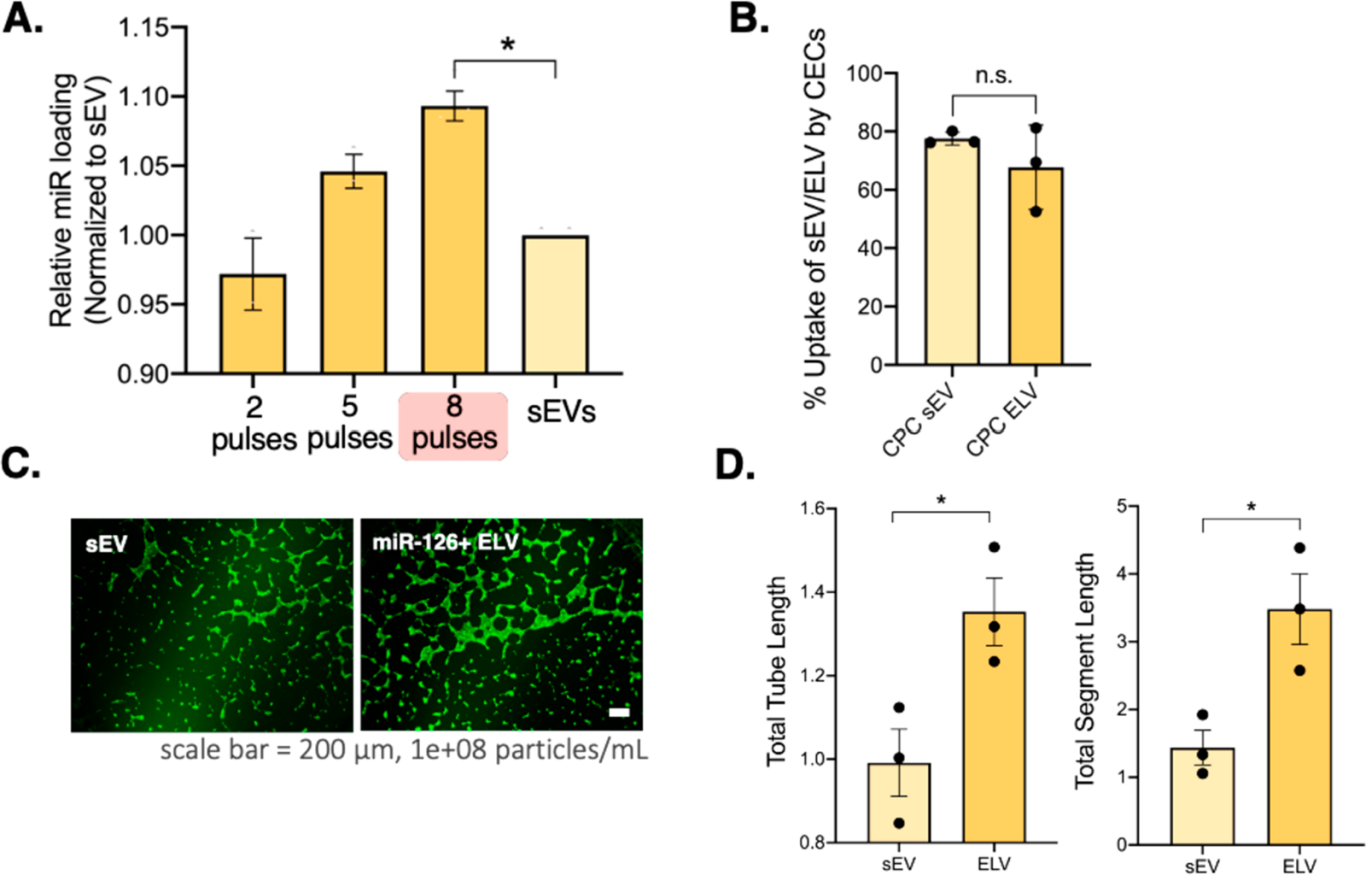
ELV cargo tunability, uptake, and induction of pro-angiogenic response when administered to CECs. (A) Tunability of miR-126 loading into ELVs by modulating the number of electroporation pulses in a square-wave electroporating setup. Red highlight shows maximum miR loading (eight pulses). (B) Uptake of calcein + sEVs and electroporated ELVs by 2D culture of CECs. (C) Calcein-AM + CECs (green) treated with electroporated miR-126+ ELVs or sEVs incubated on Matrigel form tubes overnight. (D) Quantification of angiogenic parameters of total tube length and total segment length show an increase after ELVs compared to sEV treatment. Data normalized to a negative control. Mean ± SEM. Significance was tested with one-way ANOVA with a Tukey posthoc and two-way Student’s paired *t* test. n.s. = not significant. *P < 0.05.

Having confirmed that electroporated ELVs have similar sizes and structures, we next aimed to assess the cargo tunability. Given that the ELVs in our prior published work formed by self-assembly, controlling the amount of cargo loaded was challenging.^18^ To enhance the scalability and versatility of ELVs as customized cargo-carrying vehicles, we explored the potential tunability of miR loading with electroporation. First, we optimized the sEV inherent RNA cargo depletion to provide vesicles with “cargo-free” cavities from which to synthesize the ELVs. For this, we tried four different sonication and RNase A treatments and observed that method D (maroon plot) resulted in the maximum depletion, so we proceeded with that hereforth (Supplementary Figure 1). We then explored the effect of voltage pulsing on miR-126 loading into ELVs and found that greater pulsing (up to eight pulses) allows for a significantly higher total miR per vesicle than the sEV group (Figure 2A). We therefore adopted the same cargo removal process and eight pulses during electroporation to load the cargo of choice. Finally, we confirmed the uptake of the electroporated ELVs by cardiac endothelial cells (CECs) using flow cytometry and found no significant difference in percentage uptake between sEVs and ELVs (Figure 2B).

### Electroporated miR-126+ ELVs Induce Tube Formation in CECs

To confirm the efficacy of electroporated ELVs with miR-126, we administered them to 2D cultures of CECs and compared the functional outcomes to those of sEVs. After overnight incubation on Matrigel, both ELV and sEV groups induced CEC tube formation (Figure 2C). Upon quantification of tube formation parameters, we found that electroporated ELVs significantly increase the total tube length and total segment length of the CECs compared to sEVs (Figure 2D). To confirm the working mechanism, we assessed CEC gene and protein expression (Supplementary Figures 2A and 3A). ELV treatment significantly increases PTN gene expression compared to sEV treatment, and both samples increase VEGF protein expression compared to cell-only controls. These results demonstrate that electroporated ELVs with miR-126 are more effective than sEVs in inducing angiogenic responses, despite sEVs inherently containing pro-angiogenic cargo.^7,19^

### Intramyocardial Delivery and Uptake of Vesicles

**Figure 3:**
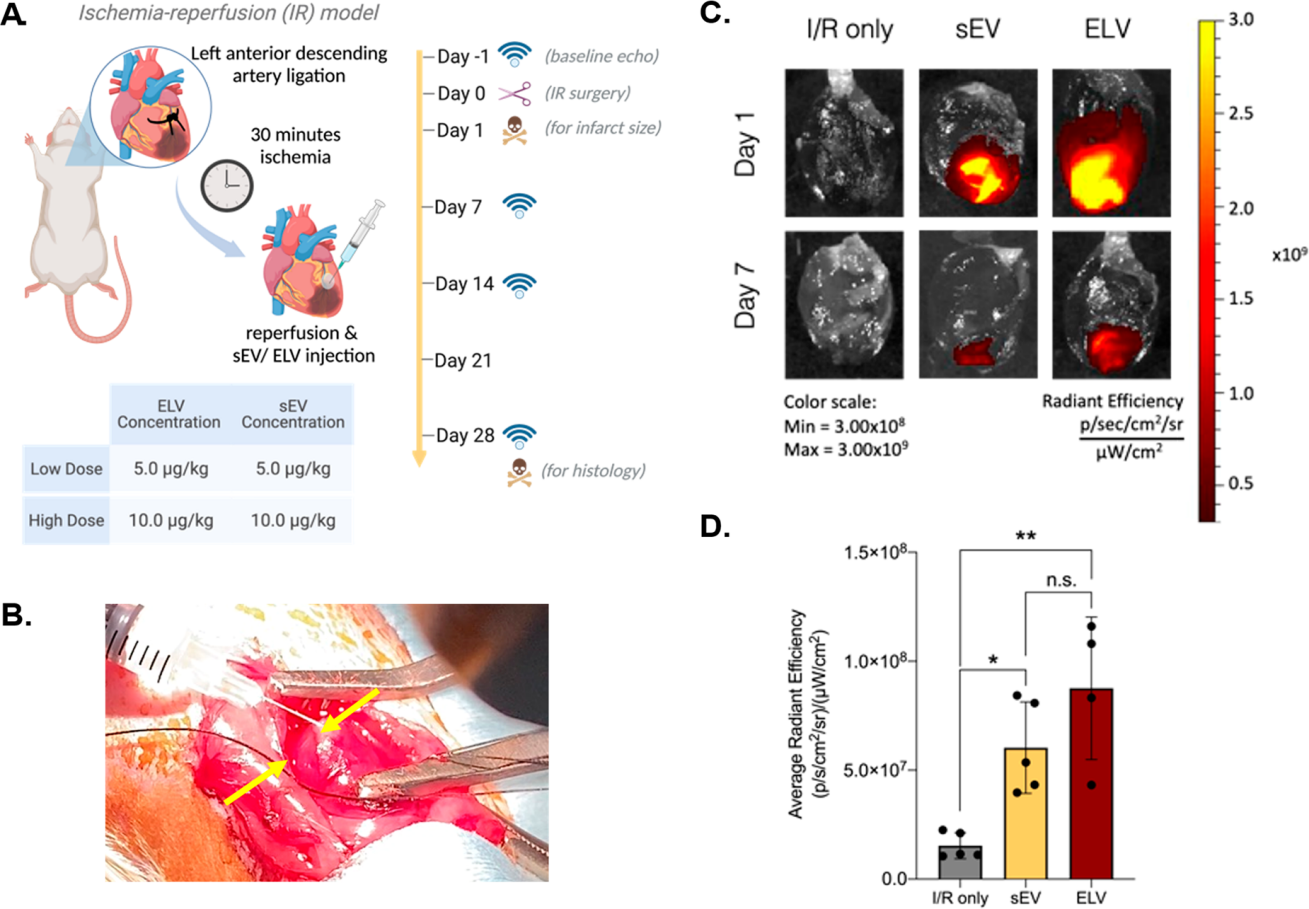
Ischemia-reperfusion animal study workflow and retention of vesicles. (A) Study workflow involves vesicle injection performed immediately after reperfusion to represent an acute model of MI. Echocardiography measurements taken at baseline and days 7, 14, and 28. Animals sacrificed 24 h post IR for infarct size assessment and at day 28 for histological analysis of myocardial tissue. (B) Representative image of intramyocardially injected sEV/ELV into the rat LV immediately after removal of the left anterior descending artery (LAD) ligation and LV reperfusion. Yellow arrows show one site of injection into the border zone; highlighted cloudy region corresponds to delivery of the sEV/ELV sample. (C) Representative imaging of in vivo DiR + sEV high and DiR+ ELV high retention at day 1 and day 7 after injection into the border zone of rat LV myocardium. (D) Quantification of DiR average radiant efficiency between I/R only, sEV high, and ELV high groups at day 7. Mean ± SEM. Significance was tested with one-way ANOVA with Tukey posthoc. n.s. = not significant, *P < 0.05, **P < 0.01.

Having ascertained the pro-angiogenic potency of miR-126+ ELVs in 2D culture, we next sought to study its role in a rat model of ischemia-reperfusion (Figure 3A). A preliminary assessment of vesicle-induced inflammatory response found reduced levels of IL-1 and IL-8 gene expression, with ELVs significantly reducing levels of IL-8 expression compared to sEVs (Supplementary Figure 2B). Further, both vesicles did not significantly increase IL-6 protein expression compared to the control (Supplementary Figure 3B). Next, to assess the effect of vesicle concentration, rats received either sEVs or miR-126+ELVs intramyocardially at a concentration of 5.0 or 10.0 μg/kg immediately after ischemia-reperfusion (IR) injury. ELV treatment was injected into the infarct border zone in 3–5 sites, and sample delivery was initially detected as a cloudy region (Figure 3B). To confirm initial retention of the sample, ELVs and sEVs prelabeled with near-infrared fluorescent dye DiIC18(7);1,1′-dioctadecyl-3,3,3′,3′-tetramethylindotricarbocyanine iodide (DiR) were administered after IR, and successful retention of vesicles in the left ventricle (LV) was assessed up to day 7 after treatment with the IVIS Spectrum imaging system. Both groups successfully retained the sample until day 7 (Figure 3C). Upon quantification at day 7, no notable differences in retention were present between sEV and ELV groups, and significant retention of both groups was present in the myocardium compared to the control (Figure 3D).

### ELVs Significantly Reduce Infarct Size in LV Myocardium after 24 h

**Figure 4:**
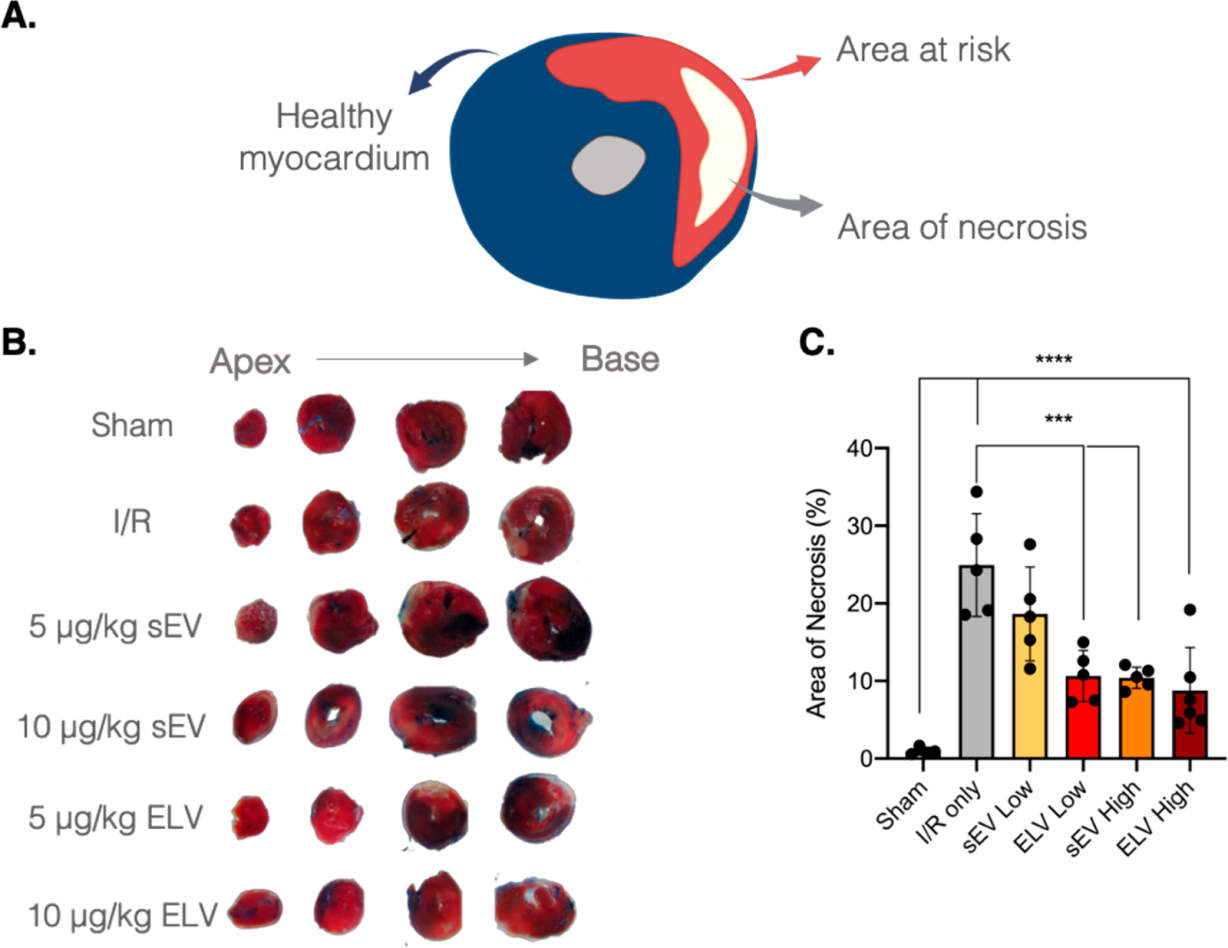
miR-126+ ELV administration reduces infarct size 24 h after vesicle administration. (A) Schematic of remote (healthy) myocardium, area at risk, and area of necrosis after TTC and Evans blue staining. (B) Representative images of myocardial tissue slices (thickness ∼2 mm) from apex (left) to base (right) stained with TTC and Evans Blue dye for infarcted and remote myocardium, respectively. (C) Quantification of percentage of necrotic tissue within the area at risk. Low = 5 μg/kg and high = 10.0 μg/kg of sEV or ELV in PBS. Mean ± SEM. Significance was tested with one-way ANOVA with Tukey posthoc. ***P < 0.001, ****P < 0.0001.

After 24 h, infarct size was determined with 2,3,5-triphenyl tetrazolium chloride (TTC) and Evans Blue dye staining. The Evans Blue stains the remote myocardium blue. The TTC stains the infarct area of risk red, and the necrosed region of the infarct bleaches white (Figure 4A). Sequential imaging of the whole myocardial tissue from the apex to base was studied to account for slight variations in the exact infarct location (Figure 4B). Upon quantification of the area of necrosis (% area of necrosis/area at risk), both ELV low and high doses and the sEV high dose significantly reduced the infarct size compared to the IR control (Figure 4C). Further, blood levels of CKMB, a marker of acute infarction, were also significantly reduced in sEV and ELV treatment groups at day 1 (Supplementary Figure 4A,B). Together, these data showed that despite the short time point, the ELV treatment groups were able to mitigate infarct progression, with a better dose profile observed for the ELV group.

### Treatment of Vesicles Improves Myocardial Function and Improvements Are More Pronounced at day 14

**Figure 5:**
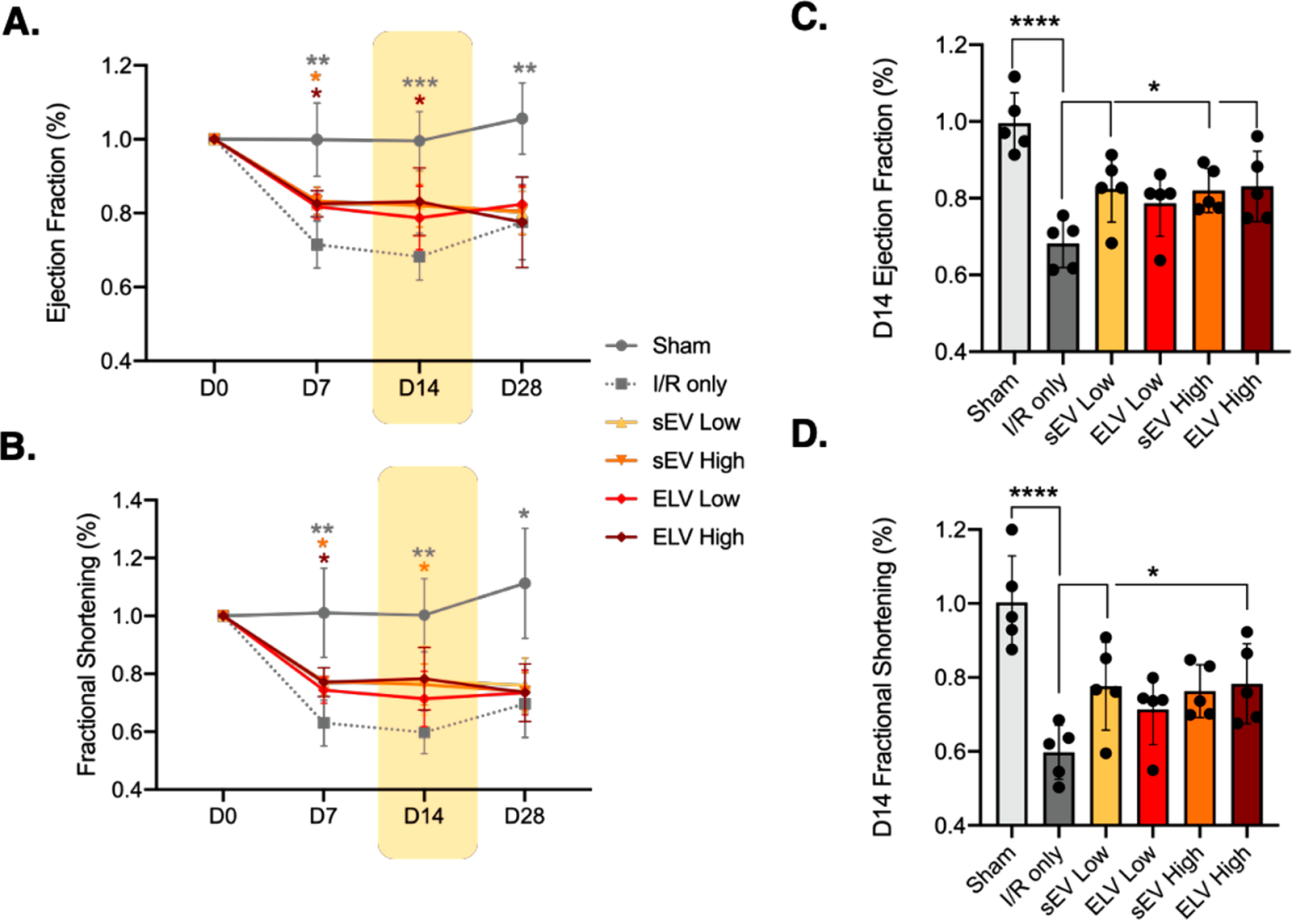
Changes in global myocardial function across 28 days after treatment with vesicles. (A) Left ventricular EF and (B) left ventricular FS in the short axis, measured at baseline and days 7, 14, and 28 after vesicle injection. Sham = gray circle, IR only = gray dotted square, sEV low = yellow triangle, sEV high = orange inverted triangle, ELV low = red diamond, ELV high = maroon diamond. (C) Differences in EF and (D) FS as compared to IR group, at day 14. Mean ± SEM. Significance was tested with two-way ANOVA with Dunnett’s post hoc for A and B and with one-way ANOVA with Dunnett’s posthoc for C and D. *P < 0.05, **P < 0.01, ***P < 0.001, ****P < 0.0001.

To determine the functional changes after treatment, left ventricular EF (Figure 5A) and FS (Figure 5B) were assessed at days 7, 14, and 28 and compared to the baseline (D0) across the left ventricular short axis. The sEV high and ELV high groups improved EF and FS compared to the IR-only control with the EF improvements sustaining until day 14 with ELV high treatment. For all of the groups, the observed functional improvements diminished again by day 28.

When focusing on day 14, both sEV groups and the ELV high group significantly improved left ventricular EF compared to the IR control (Figure 5C). In addition, the sEV low group and the ELV high group significantly improve left ventricular FS as well (Figure 5D). Given that ELVs primarily contain miR-126, unlike sEVs which contain multiple combinations of miRs, the similar global improvements observed between sEVs and ELVs are promising for our study and for ELV therapy with customized cargo loading.

### ELV Treatment Significantly Improves LV Fibrosis and Hypertrophy in the Infarct Border Zone after 28 Days

**Figure 6:**
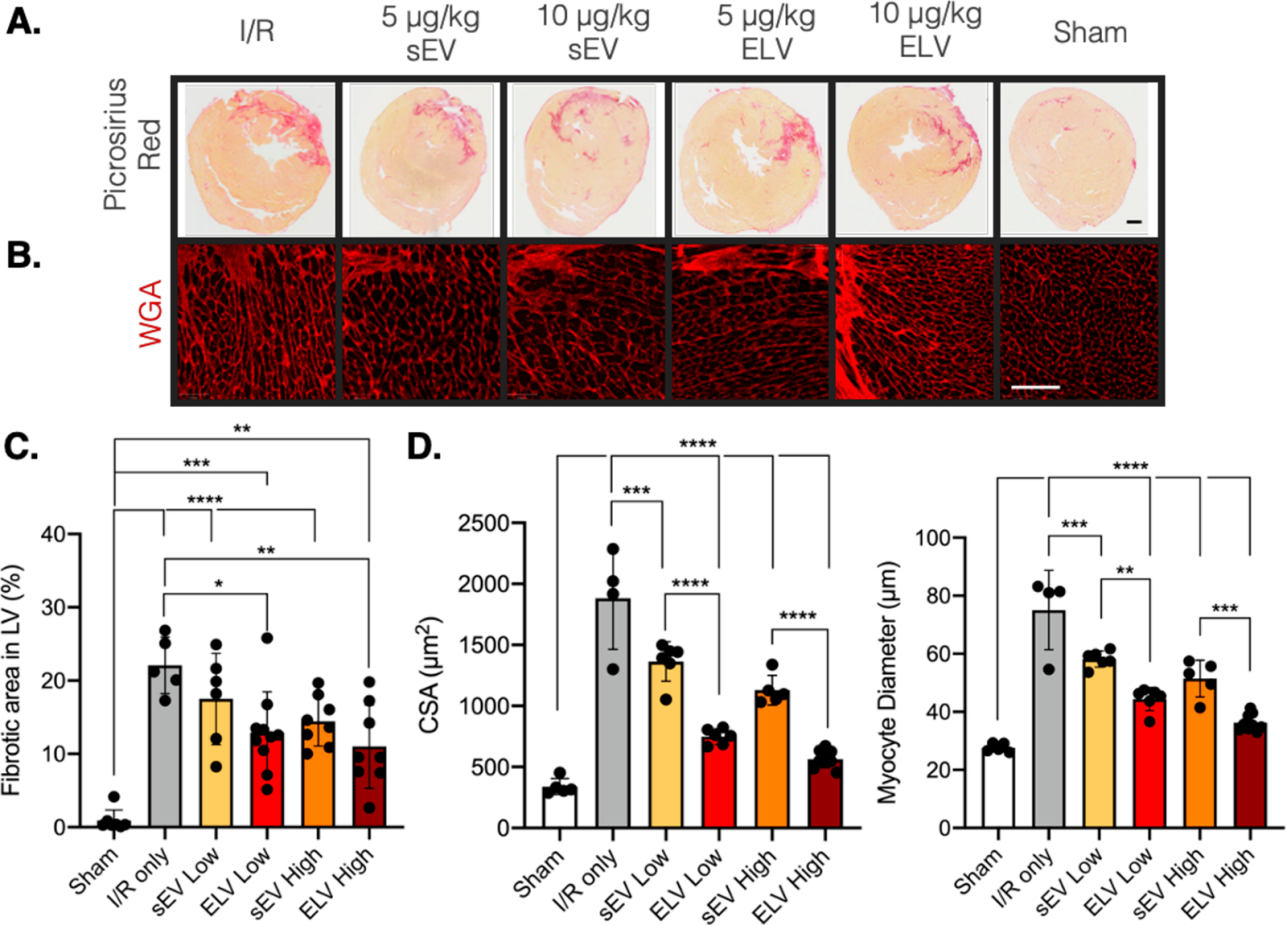
Administration of miR-126+ ELVs reduces LV fibrosis and hypertrophy 28 days after treatment. (A) Representative images of Picrosirius red-stained myocardial sections and (B) WGA + hypertrophic myocardium 28 days after vesicle treatment. (C) Quantification of fibrotic area in LV (pink) as a percentage of total LV area. (D) Quantification of average myocyte cross-sectional area and myocyte diameter as measured from WGA+ images. Low = 5 μg/kg and high = 10.0 μg/kg of ELV in PBS. Mean ± SEM. Significance was tested with one-way ANOVA with Tukey posthoc. *P < 0.05, **P < 0.01, ***P < 0.001, ****P < 0.0001. Scale bar = 1.0 mm (A) and 100 μm (B).

After establishing that vesicle-based treatments have some effect on global cardiac function, we sought to understand the role of ELV treatment at the tissue-level. Animals were sacrificed at day 28, sectioned, and stained to look at histological parameters, as described in the Experimental Methods section. Picrosirius-Red stain was used to mark connective tissue to assess LV fibrosis (Figure 6A), and wheat germ agglutinin (WGA) was used to bind cell membrane glycoproteins and in turn assess left ventricular hypertrophy (Figure 6B). Qualitatively, the representative images show a smaller fibrotic area per section and a smaller myocyte size with vesicle treatment. Upon quantification, the fibrotic area in the LV was significantly reduced by both ELV low and ELV high groups, unlike when sEVs were administered (Figure 6C). Further, the extent of improvement was more pronounced with the ELV high group (*p* < 0.01) than ELV low group (*p* < 0.05), suggesting a dose-based response. Left ventricular hypertrophy also reduced with vesicle treatment (Figure 6D), with both sEV groups and ELV groups significantly reducing myocyte cross-sectional area compared with the IR only group. Both sEV and ELV groups significantly reduced myocyte diameter compared to that of the IR only group as well. For hypertrophy, interestingly, the ELV low group significantly improved myocyte area and diameter compared to the sEV low group. Similarly, the ELV high group significantly improved both hypertrophic parameters compared to the sEV high group. This shows a clear dose-based improvement with the ELV treatment compared to sEV treatement.

### miR-126+ ELVs Increase Vessel Density and Size in the LV after 28 Days

**Figure 7:**
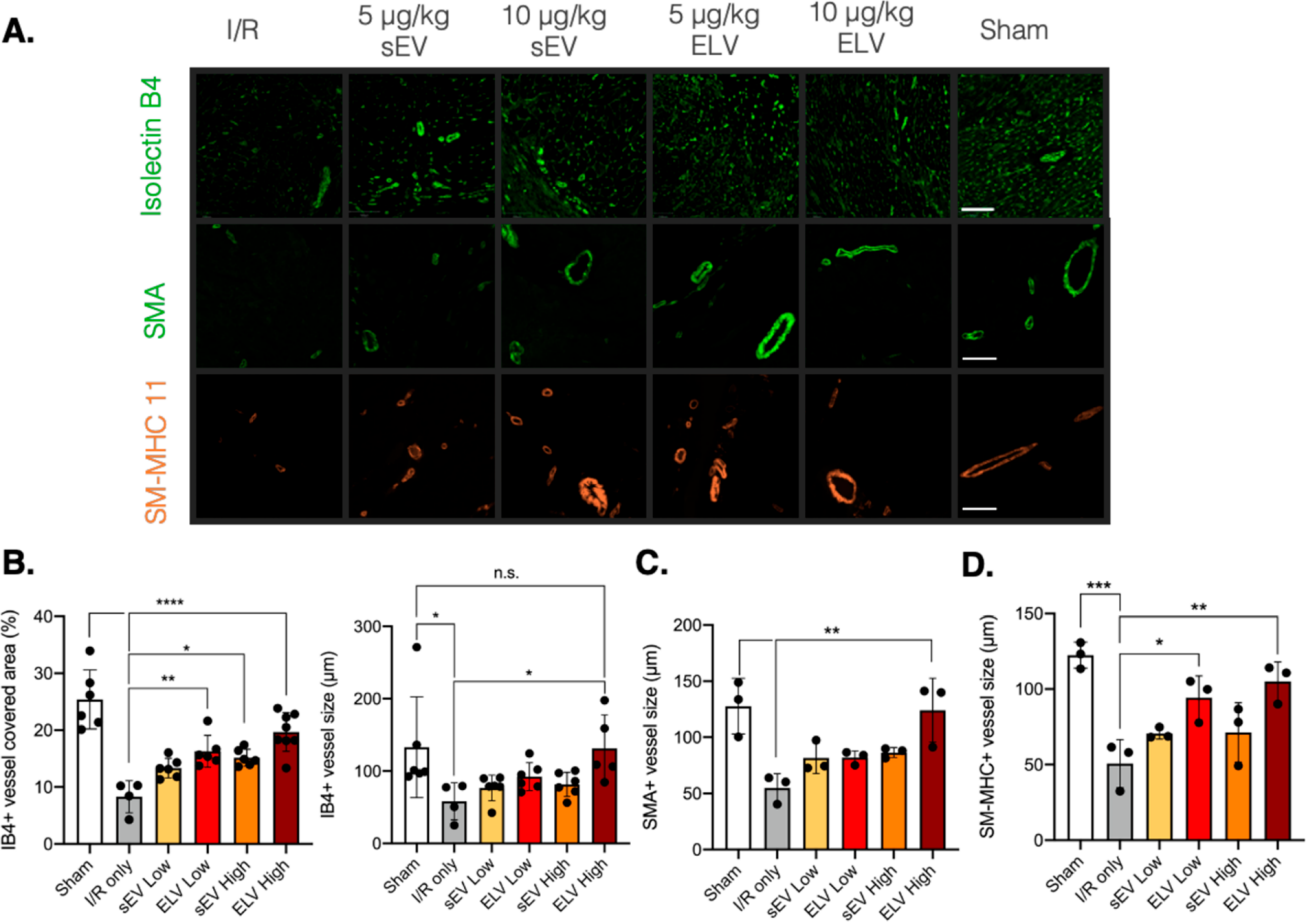
miR-126+ ELVs increase vessel formation and vessel size 28 days after treatment. (A) Representative images of isolectin-B4+, SMA+, and MHC+ vessels in ischemic myocardium 28 days after vesicle injection. (B) Quantification of isolectin-B4+ vessel area and vessel size in myocardium. (C, D) Quantification of SMA+ and MHC+ vessel size in myocardium. SMA = smooth muscle actin. SM-MHC = smooth muscle-myosin heavy chain. Significance was tested with one-way ANOVA with Tukey posthoc. n.s. = not significant, *P < 0.05, **P < 0.01, ***P < 0.001, ****P < 0.0001. Scale bar = 100 μm.

Finally, we investigated the role of ELV cargo, miR-126, in the tissue-level cardiac response. Given miR-126 is an endothelial miR which has pro-angiogenic potential *in vitro*, we chose to assess vessel-specific parameters in the LV after 28 days. Isolectin-B4 was used to detect capillaries and smooth muscle actin (SMA) and smooth muscle-myosin heavy chain (SM-MHC) 11 to detect arterioles and larger vessels (Figure 7A). Qualitatively, differences in vessel density and vessel size are noticeable between the experimental groups. For quantification, representative images were taken on the endocardial and epicardial sides of the LV corresponding to either side of the infarcted region. The sEV high group and both ELV groups significantly increased LV capillary density compared to the control (Figure 7B). The ELV high group also increased the vessel size compared to IR only and to a similar extent to the sham group. The ELV high group also significantly increased SMA stained vessel size (Figure 7C), and both ELV low and high groups increased SM-MHC labeled vessel size (Figure 7D), which indicates that the miR-126+ ELVs play a role at both the capillary and arteriole level. Combined, these results warrant the use of ELVs for selective cargo delivery and showcase that tuning ELV cargo can have significant effects *in vivo*.

## Discussion

The value and importance of sEV-based therapeutics as an alternative to cell therapy for cardiac repair is growing. Building on our previous work using thin-film hydration, here, we designed ELVs from CPC-sEVs using an electroporation method with endothelial-specific miR-126 cargo encapsulated. We show that the electroporated ELVs are of similar size and structure to sEVs and induce a pro-angiogenic response when administered to 2D CEC cultures. Further, these ELVs improve infarct size, reduce fibrosis and hypertrophy, and improve angiogenic parameters when delivered to an injured rat LV after IR injury.

To build upon our prior work, where we synthesized ELVs with a thin-film hydration process, we created electroporated ELVs for this study. We again integrated this with our sonication-based cargo depletion method to deplete inherent cargo first and allow for less variability in vesicle cargo prior to loading our cargo of choice. We chose to design our ELVs from sEVs as their complex natural membrane aids with uptake by cells and minimizes wash-out when administered in vivo.^20^ sEVs are composed of an amphiphilic lipid–protein bilayer membrane with nucleic acid–protein cargo encapsulated inside. The sEV membrane includes phospholipids, sphingomyelins, cholesterols, as well as transmembrane proteins (e.g., tetraspanins),^21^ whereas the packaged cargo is highly variable.^22^ To leverage the benefits of the sEV membrane but allow for controlled cargo, we chose to electroporate specific miR-126 into sEVs after cargo depletion. It remains to be explored, however, whether the vesicle lipid or protein composition is altered to some degree during the electroporation process.

Of the different liposome/vesicle loading methods developed recently, electroporation is considered beneficial for small noncoding RNA, which yields sufficient loading efficiency and uses minimal toxic additives.^23−25^ However, electroporation has previously been shown to aggregate siRNA and partially encapsulate RNA into the membrane instead of cytosol, thereby not having as much potency when exposed to RNase enzymes.^26^ This concern was mitigated by increasing the concentration of vesicles used for miR-loading, which was suggested to reduce aggregation, and using higher initial miR concentrations to incentivize diffusion into vesicles.^26^ Further, samples were postprocessed with RNase enzymes before ultracentrifugation, to deplete partially encapsulated miRs before downstream characterization. Another concern with electroporation of small vesicles is that their smaller diameters provide more structural stability and, therefore, higher voltages and more pulses are required to induce temporary permeabilization.^27^ This can damage the membrane and induce aggregation of nucleic acids, particularly when loading multiple cargoes.^23,28^ Here, to be mindful of this, we assessed the pulse-to-miR encapsulation and chose the least number of pulses required for miR-loading. However, in this study, we only load one miR; if multiple miR loading was desired in the future, the concern of nucleic acid aggregation should be further investigated.

*In vivo*, sEVs are valuable therapeutics for cardiac repair and recovery after MI, with similar reparative effects to the delivery of stem or progenitor cells.^6^ However, despite their therapeutic benefits, the extent of improvement is often limited with variability and inconsistencies in the observed repair and limited control over sEV cargo. Synthetic mimics mitigate some of the cargo-related variation, but they often suffer rapid flush-out and loss when delivered *in vivo*. In this work, we show that the benefits observed with CPC-derived ELVs extend to an *in vivo* rat IR model when ELVs are administered intramyocardially. We observed global echocardiographic improvements in the short axis with the sEV and ELV high groups, although these are more variable across sEVs and ELVs. At the tissue level, we find significant reduction of infarct size after 24 hours and a reduction in fibrosis and hypertrophy after 28 days. We also highlight the benefit of miR-126 cargo, with improvements observed in vessel parameters. Together, these proof-of-principle data show the potential of ELVs as a vehicle for delivery of select miRs to the myocardium after MI and warrant further study of their therapeutic benefit.

In animal studies of MI, the IR model has been suggested to be a highly representative preclinical model for investigating cardiac therapies.^29^ In patients, after suffering an acute MI, biotherapeutics or surgical interventions administered soon after the incident are desirable to maximize the cardio-protection.^30^ To recapitulate this rapid clinical response in the *in vivo* setting, there is value to administering the *in vivo* therapies right after the onset of IR. Moreover, a meta-analysis of 10 sEV therapies for acute MI in small animal models shows that most sEV therapies are delivered between 0 and 60 min after the IR.^14^ Based on these prior studies, to increase the clinical relevance of our work and for ease of intramyocardial injection while the chest cavity is opened, we chose to deliver our CPC sEVs and ELVs immediately after IR.

However, it is well established that directly after IR, the native myocardium is undergoing significant remodeling and acute repair with chemokine and pro-inflammatory cytokine release; an influx of neutrophils, monocytes, and macrophages; and the initial onset of fibrotic response and wound healing.^31^ This complex interplay of injurious and reparative events at the intramyocardial level could affect the administration of and cell response to the sEV and ELV treatments, especially when they are delivered into the infarct border zone. In our study, we found successful retention of ELV treatment after 24 hours and significant improvements at the tissue level, which suggests that despite the increase in cellular and paracrine activity in the infarcted zone, the ELVs did deliver therapeutic benefit. However, these changes did not translate to significant improvements in EF or FS after day 7 of ELV treatment, suggesting some potential disconnect between the acute and chronic changes. To explore this further, there remains value in assessing ELV delivery at later time points too. Echo-guided injections at day 14 could be conducted to separate the therapeutic benefits of the ELVs from the initial onslaught of cell and molecular response to the IR, so that we can develop a more complete understanding of the ELV’s therapeutic role after MI.

In this study, we found that there were consistently significant improvements in histological parameters at day 28, but on the global level, significant improvements were variable across time points and tapered after day 14. It should be noted that even within these improvements, no one group consistently improved global parameters over time. We suspect that the ELV treatments and miR-126 administration had tissue-level therapeutic benefit, and that was further supported by some of our cell culture mechanistic studies, but repetition with higher dosing could be required for that to translate to a global level. Moreover, the sample injection and the histological analysis were conducted in the infarct border zones, so perhaps despite cellular level repair in the immediate border, it is insufficient for a significant global improvement. In addition, the sEV and ELV dosing we used was 0.008 to 0.016 μg/μL, whereas several of the other small animal models for sEV therapies used doses from 0.2 to 2.0 μg/μL, which is 12.5 to 250 times higher dosing.^32,33^ Despite such high levels, their global functional improvements were around 3.7%, with significant heterogeneity between groups. This suggests that with even higher doses, the ELV treatments containing selective miR cargo could have a more significant reparative effect on the global scale as well.

In our ELV synthesis, we chose miR-126 as a proof-of-concept as it is an endothelial specific marker and would clearly show successful cargo loading if administered to CPC-ELVs. Beyond this, miR-126 is known to be present in endothelial progenitor cell sEVs and CD34+ stem cell sEVs and is crucial for protecting endothelial cells against injury and for sEV proangiogenic nature *in vivo* after limb ischemia.^34,35^ Similarly, miR-126 transfected MSCs also showed higher resistance to hypoxia and improved cardiac function when administered after IR.^36^ Given that miR-126 is a major regulator of angiogenesis, we chose to continue using miR-126+ ELVs for our *in vivo* studies, as well, with significant improvements detected in vessel density and size. However, to test the full scope of our ELVs, it would also be worthwhile to load other cardioprotective miRs (*e.g*., antifibrotic or anti-inflammatory miRs) and assess the cardiac responses both acute and longer term. While other groups have loaded liposomes with multiple miR cargos, we did not examine multiple miR loading in this study, and thus it remains speculative.

Vesicle administration *in vivo* can be through several methods, including open-chest intramyocardial, echo-guided intramyocardial, intravenous, subcutaneous, or intraperitoneal delivery. As we were administering our treatments immediately after IR, we chose to inject intramyocardially into the LV wall. However, to address the invasives of this approach, an intravenous injection method should be explored. One concern with non-local delivery of the ELVs would be homing to the target site, as studies have shown that sEVs delivered intravenously, subcutaneously, or intraperitoneally are rapidly cleared from circulation into the liver, kidneys, and spleen.^37,38^ However, given ELVs are engineered, there is scope to embed homing peptides (*e.g*., cardiac homing peptide,^39^ myocardium-targeting peptide,^40^ or cardiomyocyte-specific peptide^41^) onto their surface to aid with delivery and uptake into the myocardium.

Finally, another important aspect of vesicle delivery is immunomodulation and its effects on ELV efficacy. As this study involved direct targeting into the LV wall, and assessment of ELVs’ primary function was our focus, we did not extensively explore the role of the immune response (*e.g*., monocytes, cardiac tissue-resident macrophages *etc.*) on ELV potency. This would be important to study to further scale up ELV therapy. , Further assessment of tissue-level responses through TNF-α, ROS, and M1 and M2 macrophage polarization in the blood would help understand the role of the immune response on ELVs more comprehensively, as we found changes in inflammatory gene expression in vitro and in vivo. This could also warrant tailoring the ELV cargo to include specific immunomodulatory miRs to aid with function and potency.

## Conclusions

In summary, this work established that miR-126+ ELVs synthesized from CPC-dervied sEVs not only improve angiogenic parameters *in vitro* but also have significant tissue-level and marginal global level improvements when administered *in vivo* too. This work highlights the value of ELVs and the scope for using this vehicle beyond miR-126 for delivery of other cardioprotective miRs and for other cardiac applications.

## Experimental Methods

### Isolation and Culture of Human CPCs

Human CPC cells were isolated from a neonatal pediatric patient’s right atrial appendage tissue as previously described in our laboratory’s work.^15^ Briefly, CPCs were extracted through CD-117 magnetic bead sorting as per approval by the Institutional Review Board at Children’s Healthcare of Atlanta and Emory University (approval number: IRB00005500). Neonate patients were defined as those who were less than 1 week old during removal of the atrial appendage as part of a surgical procedure for a congenital heart disease. These isolated CPCs were cultured in Ham’s F-12 medium (Corning Cellgro, Corning, NY) with 10% fetal bovine serum (FBS; R&D Systems, MN), 1% penicillin-streptomycin (Thermo Fisher Scientific, MA), 1% l-glutamine, and 0.04% human fibroblast growth factor-β (hFGF-β; Sigma-Aldrich, MO). For quiescing, the CPCs were cultured in a similar Ham’s F-12 but with no serum or growth factors.

### Culture of CECs

Rat CECs were cultured as previously described.^18^ Specifically, cells were cultured in in endothelial growth medium (EGM-2 Endothelial Cell Growth Medium-2 BulletKit, Lonza, Bend, OR) supplemented with 2% FBS, 1% penicillin-streptomycin, 0.4% hFGF-β, 0.1% vascular endothelial growth factor (VEGF), 0.1% ascorbic acid, 0.1% long arginine 3 insulin-like growth factor (R3-IGF- 1), 0.1% heparin, 0.1% human epidermal growth factor (hEGF), 0.04% hydrocortisone, and 0.1% Gentamicin/Amphotericin-B (GA-1000), as per the manufacturer’s protocol. CECs were quenched in endothelial growth medium without any serum or growth factors.

### Isolation and Characterization of sEVs

2D cultures of CPCs (∼100 × 10^6^ cells) between passages 9–14 were grown up to 90% confluency as previously described.^18^ In summary, when the CPCs attained 90% confluency, the CPCs were cultured in FBS-free media and the CPC conditioned media was collected after 24 h. Next, a series of differential ultracentrifugation steps (Optima XPN-100, Beckman Coulter, Indianapolis, IN) were used to sequentially remove cells (1000 rpm for 5 min) and cell debris (15 000 rpm for 25 min) from the media, and the sEVs were finally collected as a pellet after rotation at 31 000 rpm for 114 min. These pellets were resuspended in PBS as required and stored at −80 °C until experimentation. Transmission electron microscopy (JEOL JEM-1400, Peabody, MA), NTA (NanoSight NS-300 with NTA 3.4 software, Malvern Panalytical, Malvern, UK), and Dynamic Light Scattering (DynaPro Plate Reader III, Wyatt, Santa Barbara, CA) were used to assess sEV shape, size, concentration, and polydispersity index, respectively.

### Synthesis of miR-Loaded ELVs with Electroporation

ELVs were synthesized from sEVs using a modified electroporation method. For this, sEVs were depleted of inherent cargo, and then the cargo of choice was selectively loaded. First, inherent cargo was depleted from (3–4) × 10^9^ CPC sEVs with repeated sonication cycles. For this, samples were treated with 100 μg/mL RNase A (Thermo Fisher Scientific) and sonicated at #3 with a probe-tip sonicator for 8–10 cycles: each cycle consisted of a 3 min-duration of 15 s on/off sonication, with samples kept on ice during off-cycles to minimize sample heating. Samples were then incubated for 30 min at 37 °C with constant rotation. Next, samples were treated with 40 units/20 μL of ribonuclease inhibitor (RNaseOUT, Invitrogen, Carlsbad, CA) and 1 mM DTT (Invitrogen), and the sonication step was repeated for another 8–10 cycles. Samples were then incubated for 1 h at 37 °C with constant rotation and then stored at −20 °C overnight. Samples were then electroporated with 100 pmol miR-126 (Gene Pulser Xcell, Bio-Rad, Hercules, CA) in 0.1 cm electrode gap cuvettes using 2–8 pulses (750 V square wave with 5 ms pulses). Samples were then neutralized with cold serum-free Ham’s-F-12 medium and incubated for 30 min at 37 °C with rotation followed by overnight incubation at 4 °C. Any unbound miR-126 and larger debris was removed through differential ultracentrifugation (Optima XPN-100). Larger debris was depleted after centrifugation at 1000 rpm for 5 min (Centrifuge 5810 R), smaller debris after ultracentrifugation at 15 000 rpm for 20 min (SW32Ti, Beckman Coulter), and finally the ELVs were pelleted after ultracentrifuging at 31 000 rpm for 114 min (SW32Ti, Beckman Coulter). ELVs were resuspended in PBS and stored at −80 °C until further use.

### RNA Isolation and ELV Cargo Quantification

The RNA encapsulated in ELVs and sEVs was isolated from 1.5 × 10^6^ particles of ELVs or sEVs using the miRNeasy Micro Kit (Qiagen, Germantown, MD) as per the manufacturer’s protocol. Isolated RNA total concentration was quantified (NanoDrop One, Thermo Fisher Scientific). Quantification of the miR-126 cargo presence in sEVs or ELVs was performed through a standard Real Time quantitative polymerase chain reaction (RT-qPCR) on a StepOnePlus system (Applied Biosystems, Foster City, CA).

### ELV Internalization by CECs

ELV internalization was assessed as described in prior work.^18^ More specifically, CECs were cultured until 90% confluency and then seeded at 0.05 × 10^6^ cells/well into 24 well plates precoated with 0.1% gelatin. CECs were incubated for 3–4 h for cell attachment, after which they were quiesced overnight using endothelial bare media (media without FBS or growth factors) with 1% penicillin-streptomycin. CECs were treated with calcein-stained sEVs or ELVs at 5.00 × 10^8^ particles per 1.00 × 10^6^ cells and incubated at 37 °C for 2–3 h to allow for uptake. CECs were then washed to remove unbound or partially bound vesicles, collected, and resuspended in flow buffer (2% FBS in PBS). Internalization of sEVs or ELVs by the CECs was assessed through flow cytometry (Cytek Aurora, Fremont, CA) for λ_Ex_/λ_Em_ = 495/515 nm. The negative control was CECs treated with calcein-stained ELVs or sEVs and incubated at 4 °C to inhibit uptake.

### Tube Formation Assay

CECs were cultured until 90% confluency and then quenched in endothelial bare media with 1% penicillin–streptomycin overnight. The next day, a μ-slide angiogenesis slide (IBIDI, Fitchburg, WI) was coated with 10 μL/well of Matrigel (Matrigel Matrix, Corning) as per the manufacturer’s protocol, with care to achieve an even coat. The quiesced CECs were then seeded onto the Matrigel at 10 000 cells/well and treated with either CPC sEVs or miR-126+ ELVs at 5.00 × 10^8^ vesicles per 1.00 × 10^6^ cells and incubated overnight. Quiesced CECs with no vesicle treatments were the negative control, and CECs grown in full EGM media were the positive control. Within each sample group, each well represented one technical replicate, with three replicates per group. To observe CEC tube formation, CECs were then stained with calcein-AM (Thermo Fisher Scientific) dye. Each well of the μ-slide was then imaged on a fluorescence microscope (Olympus IX71, Olympus Center Valley, PA) to assess the tube formation per well. Tube formation parameters (e.g., total tube length, total segment length etc.) were quantified with ImageJ software in pixels (Fiji, National Institutes of Health, Bethesda, MD).^42^ Specifically, the Angiogenesis Analyzer plug-in created to analyze the cellular vascular structure was used in ImageJ.

### Rat LV Ischemia-Reperfusion Model

All studies were approved by the Emory Institutional Animal Care and Use Committee (PROTO201800022). Adult male Sprague–Dawley rats were obtained from Envigo LLC. Rats 5–6 weeks old and weighing 150–175 g were used for all studies. After 1 week for acclimatization, rats were subject to ischemia-reperfusion injury as described previously.^43^ Briefly, the animals were subject to anesthesia (1–3% isoflurane), and the left anterior descending (LAD) artery was occluded for 30 min using a 4–0 silk surgical suture (Ethicon, Raritan, NJ). After occlusion, the suture is removed to initiate reperfusion injury. Two studies were performed: first, a dosage study to determine final ELV treatment dose and, next, the main study with the finalized dosages. Immediately after reperfusion, animals were subjected to one of the treatment groups (refer to next section). Sham rats underwent the same procedure except for ligation of the LAD. After completion of surgery, the animals were housed at the Emory University Animal Research Facility.

### sEV or ELV Treatment in Vivo

Administration of all treatments was conducted in a randomized and blinded manner. Treatment groups included sham, IR-only (saline treatment), sEV low, sEV high, ELV low, and ELV high. For the study, sEVs or miR-126+ ELVs at 5.0 or 10 μg/kg were administered in 150 μL of saline or saline only. Treatments were injected into three to five areas of the ischemic border zone with a 30-gauge insulin syringe (Ultrafine needle, BD, Franklin Lakes, NJ).

### Infarct Size Staining and Quantification

Twenty-four hours after IR surgery, each animal’s myocardium was accessed again through the initial surgical incision, and the LAD was religated with a suture left in place during the initial IR surgery. The LV wall was then injected with 50–80 μL of Evans blue dye, adjusted for heart size, to perfuse the remote myocardium. The heart was then resected and washed in a Petri dish with PBS to remove excess Evans blue dye and blood. The heart was then wrapped in Saran wrap and stored at −20 °C to −80 °C to solidify the tissue. After solidification, the heart was cut into 1.5–2 mm slices along the short axis with a cold blade atop a prefrozen granite tile. Cut cross sections were then incubated with freshly made 1% TTC in 0.9% NaCl for 15 min at 37 °C, under constant rotation to expose the area at risk and area of necrosis.^44^ The cross sections were then fixed in 10% neutral-buffered formalin for up to 90 min and stored in PBS at 4 °C until imaging. Samples were imaged using a Nikon DS600 camera, and the area of necrosis (white region), area at risk (red region), and remote myocardium (deep blue region) were outlined and quantified using ImageJ software.^42^ Area at risk was noted as a percentage of the whole heart, and area of necrosis was noted as a percentage of the area at risk.

### Echocardiography and Hemodynamic Analysis

Rats were anesthetized with inhaled 2–4% isoflurane with 100% oxygen and subject to echocardiography prior to surgery (baseline), at day 7, day 14, and day 28 postsurgery with a high frequency transducer. M-mode short axis views were taken by using a Vevo 3100 digital high-frequency preclinical ultrasound system (FujiFilm Visualsonics, Loveland, CO) for global hemodynamic values. An average of three to six consecutive cardiac cycles were taken for each measurement, and this was taken three times per animal in a blinded manner. Data were analyzed using VevoLAB software.

### Histological Tissue Sectioning and Staining

At day 28, after the completion of the study, animals were sacrificed and the hearts resected. The hearts were fixed in 10% formalin overnight and then transferred to 30% sucrose buffer for 2–3 days until the sucrose penetrated through the tissue (and the hearts “sank”). The hearts were then embedded in an optimal cutting temperature (OCT) compound (Tissue-Tek, Fisher Scientific) and stored at −80 °C. For histological analysis, embedded hearts were sectioned into 8 μm thick slices with the Leica CM1520 Cryostat and immunostained with isolectin-B4 (Vector Laboratories FL-1201) for capillary assessment, WGA (Vector Laboratories, Rhodamine-labeled RL10225) for hypertrophy assessment, and alpha-SMA (Cy3-labeled C6198 Millipore Sigma) or SM-MHC-11 (Ab50967, Abcam; Alexa Fluor 647, 560400, BD Biosciences) for arteriole and vessel assessment. The sections were also stained with picrosirius red connective tissue stain (Ab150681, Abcam) to assess myocardial fibrosis. All stained sections were imaged by the Cancer Tissue Pathology Core (Winship Cancer Institute) at 20× immunofluorescence or bright-field microscopy, as required.

### Statistical Analysis

GraphPad PRISM 8 software (GraphPad, San Diego, CA) was used to complete all statistical analyses for this study. Specific details pertaining to each analysis are described in the corresponding figure captions.

## Supporting Information Available

Data to show sEV internal cargo depletion using four different cargo removal methodologies. Supplemental Figure 1: Optimization of CPC sEV cargo depletion. Supplemental Figure 2: Relative gene expression of CECs and macrophages after treatment with sEVs and miR-126+ELVs. Supplemental Figure 3: Protein expression of CECs and macrophages after treatment with sEVs and miR-126+ELVs. Supplemental Figure 4. Assessment of cardiomyocyte death (PDF)

## Abbreviations

MI: myocardial infarction
CPC: ckit+ progenitor cell
MSC: mesenchymal stromal cell
sEV: small extracellular vesicle
EF: ejection fraction
FS: fractional shorterning
microRNA: miR
ELV: small extracellular vesicle-like vehicle
NTA: nanoparticle tracking analysis
CEC: cardiac endothelial cell
IR: ischemia-reperfusion
DiR: DiIC18(7);1,1′-dioctadecyl-3,3,3′,3′-tetramethylindotricarbocyanine iodide
LV: left ventricle
TTC: 2,3,5-triphenyl tetrazolium chloride
SMA: smooth muscle actin
SM-MHC: smooth muscle–myosin heavy chain
