# Supplementary material for: Customized Loading of microRNA-126 to Small Extracellular Vesicle-Derived Vehicles Improves Cardiac Function after Myocardial Infarction
Source: ACS Nano. 2023 Sep 16;17(20):19613–24. doi: 10.1021/acsnano.3c01534 (PMC10604069; doi:10.1021/acsnano.3c01534)
Supplement: Supplementary file 1 — nn3c01534_si_001.pdf [file nn3c01534_si_001.pdf]

Customized loading of microRNA-126 to small extracellular vesicle-derived vehicles improves cardiac function after myocardial infarction

Sruti Bheri[1], Milton E. Brown[1], Hyun-Ji Park[1,2], Olga Brazhkina[1], Felipe Takaesu[1,3], Michael E. Davis[1,4]*

[1]Wallace H. Coulter Department of Biomedical Engineering, Georgia Institute of Technology & Emory University, Atlanta, GA, USA, 30332

[2]Department of Molecular Science and Technology, Ajou University, Suwon, Korea 16499

[3]Biochemistry, Cell and Developmental Biology Graduate Training Program, Graduate Division of Biological and Biomedical Sciences, Laney Graduate School, Emory University, Atlanta, GA, USA, 30332

[4]Children's Heart Research & Outcomes (HeRO) Center, Children's Healthcare of Atlanta & Emory University, Atlanta, GA, USA, 30322

[*]Corresponding Author: michael.davis@bme.gatech.edu

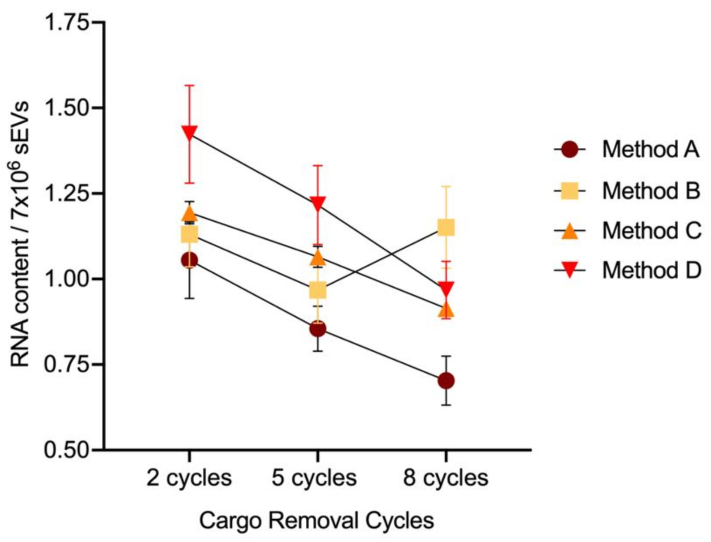

Process	Method A	B	C	D
Sonication	✓	✓	✓	
RNAse A Treatment + Sonication		✓	✓	✓
RNAseA incubation	✓	✓	✓	✓
RNAseOUT Treatment + Sonication			✓	✓
RNAseOUT incubation	✓	✓	✓	✓

Supplemental Figure 1: Optimization of CPC sEV cargo depletion. Total RNA cargo present in CPC sEVs quantified after 2, 5 and 8 sonication cycles using Methods A, B, C or D. Increased sonication cycling increases cargo depletion with largest RNA cargo depletion corresponding to the steepest slope (Method D). Total RNA content quantified using NanoDrop spectrophotometer and normalized to total number of sEVs. n=3. Mean±SEM

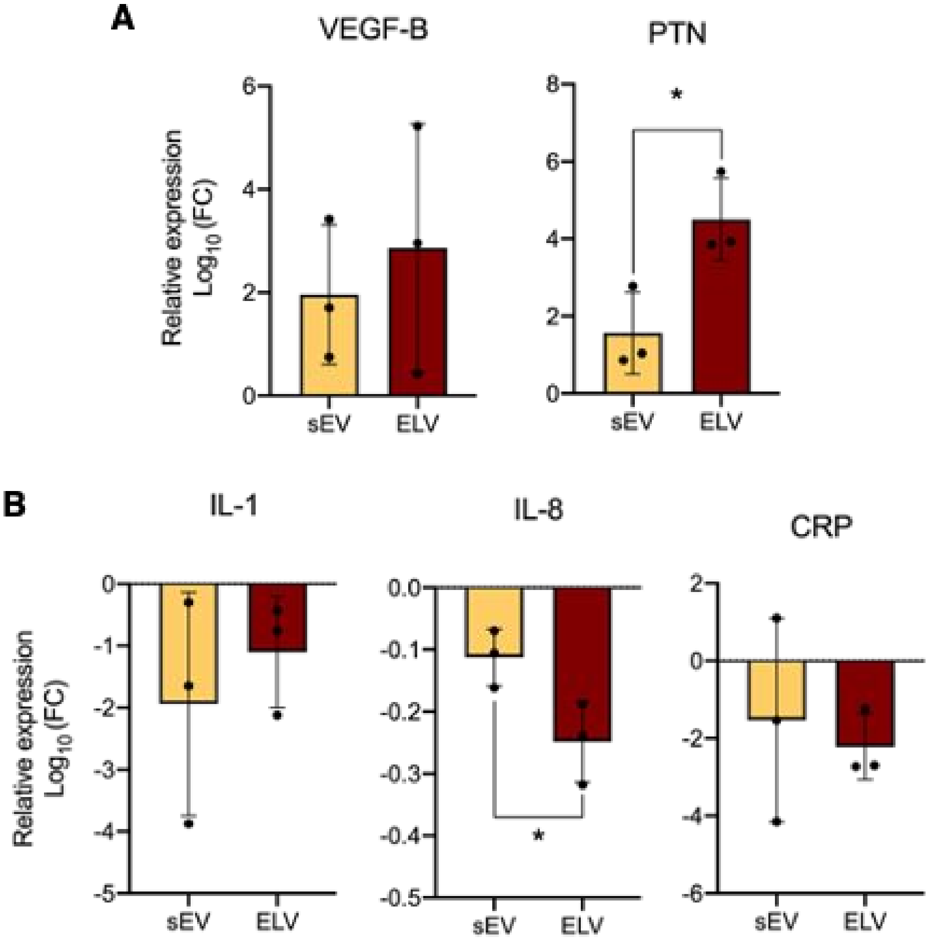

Supplemental Figure 2: Relative gene expression of CECs and macrophages after treatment with sEVs and miR-126+ELVs. (A) Gene markers for angiogenesis show increased expression of VEGF-B and PTN in sEVs and ELVs with significantly more PTN expressed in ELVs. (B) Gene markers for inflammation show decreased expression of IL-1, IL-8 in both sEV and ELV groups with ELV treatment significantly reducing IL-8 expression. ELV group also has less variability in CRP reduction. All data collected with RT-qPCR and normalized to quiesced cell-only controls. Mean±SEM. Significance was tested with two-way Student's unpaired *t* test. *$P<0.05$. VEGF: vascular endothelial growth factor; PTN: pleiotrophin; IL-1/IL-8: interleukin-1/ -8; CRP: C-reactive protein.

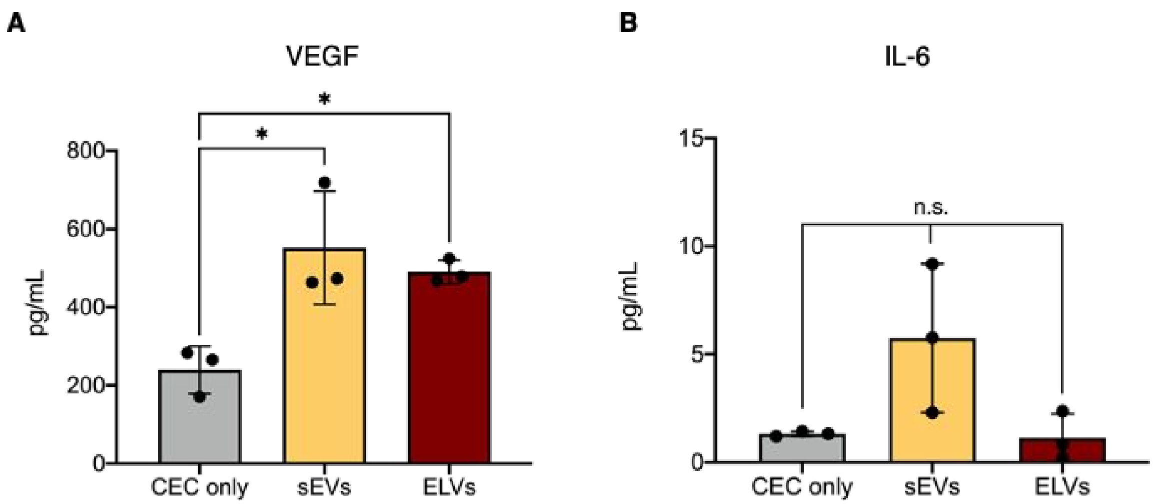

Supplemental Figure 3: Protein expression of CECs and macrophages after treatment with sEVs and miR-126+ELVs. (A) VEGF protein expression in CECs. Significant increase in VEGF in CECs treated with sEVs or ELVs. (B) IL-6 protein expression in macrophages. No significant difference in IL-6 expression between groups. All data collected with solid phase sandwich ELISA assay. Mean±SEM. Significance was tested with one-way ANOVA with Tukey post-hoc. n.s. = not significant. *P<0.05. VEGF: vascular endothelial growth factor IL-6: interleukin-6.

A

CKMB

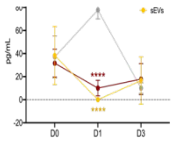

B

CKMB Day 1

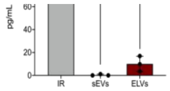

Supplemental Figure 4: Assessment of cardiomyocyte death. CKMB levels in the rat blood were measured as an indicator of acute myocardial infarction. (A) CKMB levels from Day 0 (immediately post-surgery) until Day 3. (B) CKMB levels compared at Day 1 shows significant reduction in CKMB levels for sEVs and ELVs. All data collected with solid phase sandwich ELISA assay. Mean±SEM. Significance was tested with two-way ANOVA and one-way ANOVA with Tukey post-hoc. ****P<0.0001. CKMB: Creatine Kinase MB.
